# Next Generation Sequencing Identifies Subgroups of Patients With Triple Negative Primary Thrombocytosis With Different Clinical Thrombotic Outcomes

**DOI:** 10.1111/ijlh.14476

**Published:** 2025-04-07

**Authors:** Valentina Sangiorgio, Federica Mottadelli, Fabio Pagni, Fabrizio Cavalca, Giovanni Cazzaniga, Martina Venegoni, Carlo Gambacorti‐Passerini, Rocco Piazza, Elena Maria Elli

**Affiliations:** ^1^ School of Medicine and Surgery University of Milan‐Bicocca Monza Italy; ^2^ Tettamanti Center Fondazione IRCCS San Gerardo Dei Tintori Monza Italy; ^3^ Division of Hematology and Bone Marrow Unit Fondazione IRCCS San Gerardo Dei Tintori Monza Italy

**Keywords:** bone marrow, essential thrombocythemia, molecular genetics, myeloproliferative syndromes, thrombosis

## Abstract

**Introduction:**

The majority of patients with essential thrombocythemia (ET) show somatic mutations of *JAK2, CALR*, or *MPL*. Around 10% of cases lack these mutations (“triple negative” ET, TN‐ET). Additionally, some patients with *bona fide* “primary thrombocytosis” (PT) [i.e., high platelet (PLT)‐ count with no apparent underlying causes] do not fulfill the histologic criteria of ET. In this context, Next Generation Sequencing (NGS) can provide evidence of clonality and identify patients with different clinical behaviors.

**Methods:**

We conducted a retro‐prospective analysis of 39 patients with TN‐PT and correlated the clinical and pathologic features with the molecular findings.

**Results:**

Bone marrow histopathological features were consistent with ET in 60% of cases. After a mean follow up of 11.1 years, no cases of secondary myelofibrosis nor acute leukemia were observed. We reported 15 thrombotic events (TEs) in 10 (25.6%) patients. Considering mutations with a variant frequency ≥ 5%, 15.4% of patients showed at least one mutation (“NGS‐positive”); the remaining had no mutations (“NGS‐negative”). NGS status predicted the incidence of TEs: NGS‐positive patients experienced a significantly higher rate of TEs compared to NGS‐negative patients (66.6% vs. 18.2%, respectively; *p =* 0.01).

**Conclusion:**

NGS status represents an adjunctive risk factor for thrombosis in TN‐PT and provides useful clinical information.

## 1 Introduction

Chronic thrombocytosis is defined by a persistent (> 6 months) raise in platelet (PLT) count ≥ 450 × 10^9^/L. In daily practice, this is more commonly secondary to a plethora of conditions ranging from iron deficiency to infections, inflammation, autoimmune diseases, trauma, hemolysis, metastatic cancer or secondary to splenectomy [1]. Primary thrombocytosis (PT) occurs in a minority of the cases as a manifestation of myeloid malignancies, mostly myeloproliferative neoplasms (MPN) and myelodysplastic/myeloproliferative neoplasms (MDS/MPN). Also, rare cases of familial inherited thrombocytosis are reported [2]. Among the myeloid malignancies, essential thrombocythemia (ET) is the most frequent cause of PT, characterized by a relatively isolated increase in PLT count, a high risk of vascular events including thromboses and hemorrhages and a potential evolution into secondary myelofibrosis and overt blast phase (BP) [3].

The diagnostic workup of isolated thrombocytosis starts with the investigation of family history and the exclusion of secondary etiologies [4]. Once these are excluded, the histologic examination of bone marrow trephine (BMT) biopsies and the search for the classical MPN driver mutations (involving *JAK2, CALR* and *MPL* genes) become mandatory to investigate a possible primary condition. Around 10%–20% of patients with bona fide ET are negative for the three driver mutations (so called “triple‐negative” ET) [5]. According to the current WHO Classification, the diagnosis of ET requires the typical histological features together with the identification of a common MPN driver mutation. In the absence of the latter, a diagnosis of ET can still be performed provided the presence of other clonal marker/s or the exclusion of other causes of secondary thrombocytosis [6]. Nevertheless a number of patients with a “triple‐negative” molecular signature do not display the characteristic histological features of ET nor of other MPN, raising the question of their most appropriate therapeutic management. Indeed, the main clinical goal in ET is the prevention of vascular events, a probability estimated using the International Prognostic Score of Thrombosis for Essential Thrombocythemia (IPSET) score, which stratifies patients into “low”, “intermediate” or “high” risk [7, 8]. While antiplatelet therapy is generally suggested from low risk upwards, a cytoreductive treatment is recommended only for intermediate and high‐risk patients [9, 10]. However, when the diagnosis of ET is uncertain, the benefit of a cytoreductive therapy is not known.

In the last decade, Next Generation Sequencing (NGS) has been established as a reliable diagnostic tool to investigate the molecular landscape of neoplastic diseases, including MPN. In the context of TN‐PT, NGS can identify non‐canonical driver mutations, thus ruling out secondary thrombocytosis [6], or it can recognize possible germline variants in genes involved in myeloid neoplasms [4].

NGS findings may also carry prognostic implications. In Primary Myelofibrosis (PMF), patients with a TN signature experience a more aggressive clinical phenotype [11, 12, 13]. TN‐ET contains a more heterogeneous group of patients with different outcomes in terms of thrombosis, survival, and disease evolution [4, 5].

Lastly, NGS can quantify the “mutational burden” for each variant through the prospective monitoring of the associated VAF. This quantification can theoretically allow a more accurate monitoring, especially in the era of drugs such as pegylated interferon or JAK2 inhibitors, which can induce a “molecular response” [14, 15].

The aim of the present study was to analyze the clinical and laboratory parameters alongside the bone marrow histopathological findings in a real‐world cohort of TN‐PT, as well as to determine their molecular profile using a diagnostically oriented NGS panel to identify possible differences in clinical outcome.

## 2 Material and Methods

### 2.1 Study Design and Statistical Analysis

We conducted a retro‐prospective analysis of adult patients with history of chronic persistent TN‐PT (PLT count, ≥ 450 × 109/L for > 6 months), followed at the Hematology Unit of the Fondazione IRCCS San Gerardo Hospital in Monza, Italy between 2001 and 2023. Inclusion criteria were the availability of demographic, clinical, hematologic data at diagnosis and at least one granulocyte DNA sample from peripheral blood (PB) (collected at the time of the MPN driver mutational screening) to perform NGS analysis. The presence of a BMT sample for the histopathological diagnosis was desirable, but not mandatory for inclusion. All patients presented a “triple negative phenotype” (i.e., absence of driver JAK2, CALR and MPL mutations) and did not fulfill the diagnostic criteria of other MPN or MDS/MPN [6]. Secondary causes of thrombocytosis were excluded by the mean of clinical and laboratory analyses.

For each patient we collected information on demographic, clinical, therapeutic, laboratory features, and clinical outcome including the incidence of thrombosis, hemorrhagic events, and evolution into secondary myelofibrosis (MF), post‐ET‐MF, or BP. As main cardiovascular risk factors, we considered the following: arterial hypertension, diabetes mellitus, hyperlipidemia, and smoking attitude. Thromboses were defined according to the International Classification of Diseases (9th revision) and graded according to the Common Terminology Criteria for Adverse Events (CTCAE) v4.0. Diagnosis of post‐ET MF was made in accordance with the International Working Group for Myeloproliferative Neoplasms Research and Treatment (IWGMRT) criteria [16]. Diagnosis of BP was made with a 20% BM or PB blast threshold. Patients were treated according to current European Leukemia Net guidelines [17].

Regarding the analytic approach, comparisons of quantitative variables between groups of patients were carried out using the Wilcoxon–Mann–Whitney rank‐sum test, and associations between categorical variables were tested using the χ^2^ test. Thrombosis‐free survival (TFS) was defined as the time from TN‐PT diagnosis to the first thrombotic event. TFS curves were estimated by the Kaplan–Meier method; the log‐rank test was used to compare curves in different NGS groups.

An informed consent specific to the current study was obtained for all the enrolled patients for the clinical and molecular (i.e., somatic) data from blood or bone marrow samples. This consent did not include the possibility to perform tumor‐normal paired testing and consequently, for some variants, the presumed germline nature could not be assessed.

### 2.2 Bone Marrow Histopathological Evaluation

BMT biopsies were independently reviewed by two pathologists with a special experience in hematopathology (VS, FP). In particular, sections were stained with Hematoxylin and Eosin, Giemsa, and Gomori's silver stain, the latter for the evaluation of BM fibrosis. Morphologic parameters were assessed and reported as required by the WHO Classification—2022 Edition [6].

### 2.3 Molecular Analysis

Genomic DNA was extracted from granulocytes of patients enrolled in the study and tested for driver mutations in *JAK2, CALR*, and *MPL* genes. V617F mutation in *JAK2* was tested with ASO‐PCR [18]. Mutations in exon 12 of *JAK2*, exon 9 of *CALR*, and exon 10 of *MPL* were tested with Sanger sequencing. All patients enrolled had a NGS analyses done on genomic DNA extracted from whole PB. NGS analysis was performed using the Illumina MiSeqTM platform and the Sophia Myeloid Solution gene panel, which covers the coding sequences and the flanking regions of the 30 most clinically relevant genes associated with myeloid neoplasms (Myeloid Solutions Panel [Table S1]. SOPHiA Genetics, Saint Sulpice, Switzerland). Variant calling was carried out using Sophia DDM software, and each variant was pre‐classified according to its expected pathogenicity (SOPHiA DDM Platform. SOPHiA Genetics, Saint Sulpice, Switzerland) (Table S2).

Initially for a comprehensive description, we considered pathogenic/likely pathogenic mutations with VAF > 0.5% [19]. For the subsequent correlations, a cut‐off of ≥ 5% was utilized. Pathogenic/likely pathogenic variants with a VAF approaching 50% were excluded as their germline nature could not be excluded. Information on conventional karyotype was collected whenever available.

## 3 Results

### 3.1 Clinical and Pathologic Findings

In total 39 patients were enrolled. The main clinical and laboratory findings are shown in Table 1. In particular, the mean age at diagnosis was 47 years (range: 13–82). The mean PLT count was 695 × 10^9^/L (range: 481–1838). Hemoglobin, white blood cell count and lactate dehydrogenase were all within normal limits. The IPSET score was low (score 0–1) in 25 patients (64.1%), intermediate (score 2) in 10 patients (25.6%) and high (score > 2) in 4 patients (10.3%).

**TABLE 1:** Main clinical and laboratory findings in a cohort of 39 patients with triple‐negative primary thrombocytosis, with stratification for NGS status.

	Overall cohort: 39	NGS positive: 6	NGS negative: 33	*p*
Demographic parameter				
Male/Female, number	12/27	1/5	11/22	ns
Age at diagnosis, mean (range)	47 (13–82)	65 (42–82)	43 (13–80)	0.01
Laboratory values				
PLT count ×109/L, mean (range)	695 (481–1838)	510 (481–535)	728 (486–1838)	ns
Hb (g/dL), mean (range)	14 (10.9–16.8)	13.9 (12.6–15.7)	13.8 (10.9–16.8)	ns
WBC count ×109/L, mean (range)	8 (4.16–12.7)	7.0 (4.75–11.6)	7.6 (4.16–12.7)	ns
LDH U/L, mean (range)	221 (145–387)	179 (145–228)	232 (172–387)	ns
Cytogenetic abnormalities, number of patients (%)	0 (0)	0 (0)	0 (0)	ns
Clinical findings				
Splenomegaly, number of patients (%)	0 (0)	0 (0)	0 (0)	ns
Bleeding events, number of patients (%)	0 (0)	0 (0)	0 (0)	ns
Thrombotic events, total number	15	7	9	
Thrombotic events, number of patients (%)	10 (25.6%)	4 (66.6)	6 (18.2)	0.01
IPSET‐low, number of patients (%)	25 (64.1)	3 (50)	22 (66.6)	ns
IPSET‐intermediate, number of patients (%)	10 (25.6)	1 (16.7)	9 (27.3)	ns
IPSET‐high, number of patients (%)	4 (10.3)	2 (33.3)	2 (6.0)	0.04
Smoking, number of patients (%)	4 (10.3)	1 (16.6)	3 (10)	ns
Diabetes, number of patients (%)	0 (0)	0 (0)	0 (0)	ns
Hypertension, number of patients (%)	13 (33.3)	3 (50)	10 (30.3)	ns
Dyslipidemia, number of patients (%)	12 (30.8)	3 (50)	9 (27.3)	ns
Histological findings^a^				
Consistent with ET, number of patients (%)	21 (60)	3 (60)	18 (60)	ns
Medical therapy				
Antiplatelet therapy, number of patients (%)	36 (92.3)	5 (83.3)	31 (93.9)	ns
Cytoreductive therapy, number of patients (%)	21 (53.9)	3 (50)	18 (54.5)	ns

*Note*: Data are shown within the entire cohort and according to NGS status (i.e., NGS positive vs. negative patients). Only pathogenic/likely pathogenic variants with VAF ≥ 5% have been considered. A *p*. value ≤ 0.05 was considered significant.

Abbreviations: F, females; g/dL, grams per deciliter; Hb, hemoglobin; IPSET, International Prognostic Score of Thrombosis for Essential Thrombocythemia; LDH, lactate dehydrogenase; M, males; mU/mL, milliunits per milliliter; NGS‐neg, Next Generation sequencing negative patients; NGS‐pos, Next Generation sequencing positive patients; ns, not significant; PLT, platelet count; U/L, unit per liter; yrs, year.

^a^ Bone marrow was available for histopathological revision for 35 patients of the overall cohort.

**FIGURE 1:**
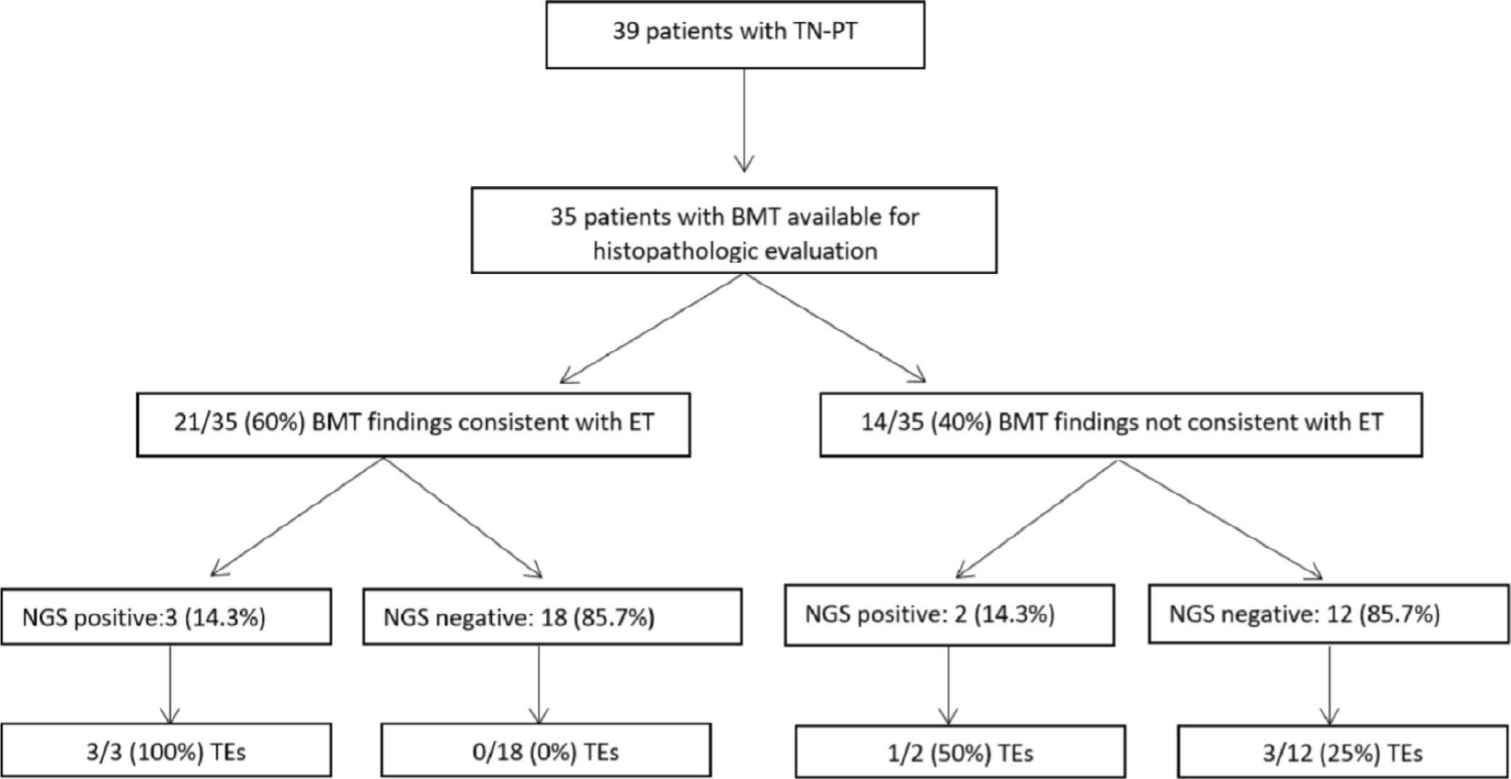
Diagnostic workflow in the studied cohort of 39 patients with triple‐negative primary thrombocytosis. In this illustration, NGS variants are filtered at a VAF of ≥ 5%. Of note, one NGS positive patient did not have a BMT sample performed within 10 years from the diagnosis of TN‐PT. BMT, Bone marrow trephine biopsy; NGS, Next generation sequencing findings; TN‐PT, Triple‐negative primary thrombocytosis; TEs, Thrombotic events.

A BMT biopsy was available for 35 patients (89.7%), in 28 of them (80%) within the first 2 years from the initial clinical diagnosis. The diagnostic workflow for the studied cohort is summarized in Figure 1. After the histologic review, the microscopic findings were consistent with ET in 21 cases (60%). The remaining 14 patients (40%) did not fulfill the histopathological criteria of ET; albeit, some degree of megakaryocytic hyperplasia could be noted in some cases.

Lastly, 7 patients (18%) presented with a PLT count ≥ 900 × 10^9^/L (range: 918–1838). All of them had a low IPSET score and a BMT consistent with ET in 6 cases.

### 3.2 Therapeutic Management and Follow up

In total, 36 patients (92.3%) received an anti‐platelet therapy, mainly acetylsalicylic acid 100 mg daily; 3 patients who did not receive an anti‐platelet therapy were young (< 40 years old), had a low IPSET (score 0) and normal morphologic features at BM biopsy. Twenty‐one patients (53.9%) required a cytoreductive therapy: hydroxycarbamide was used in 17 cases (80.9%); 4 patients (19.1%) received anagrelide.

A total of 15 TEs occurred overall in 10 patients (25.6%); of them, 8 TEs were reported at diagnosis or within 2 years before onset of thrombocytosis, while 7 TEs occurred during follow‐up. The thrombotic rate of the entire cohort was of 1.62 events per 100 patient‐years.

The majority of TEs (86.6%) were arterial thrombosis: 6 transient ischemic attack (TIA) or stroke, 5 acute myocardial infarctions, 1 episode of preeclampsia, 1 carotid artery thrombosis. An episode of superficial vein thrombosis and 1 pulmonary embolism were reported in 2 patients, respectively.

Regarding the hemorrhagic events, one patient had recurrent episodes of epistaxis. Otherwise, relevant hemorrhagic episodes occurred neither at presentation nor during follow up.

After a mean follow up of 11.1 years (range 1.9–33.6), none of the patients in the cohort developed secondary MF or progression into BP.

### 3.3 Molecular Results and Clinical Implications

**FIGURE 2:**
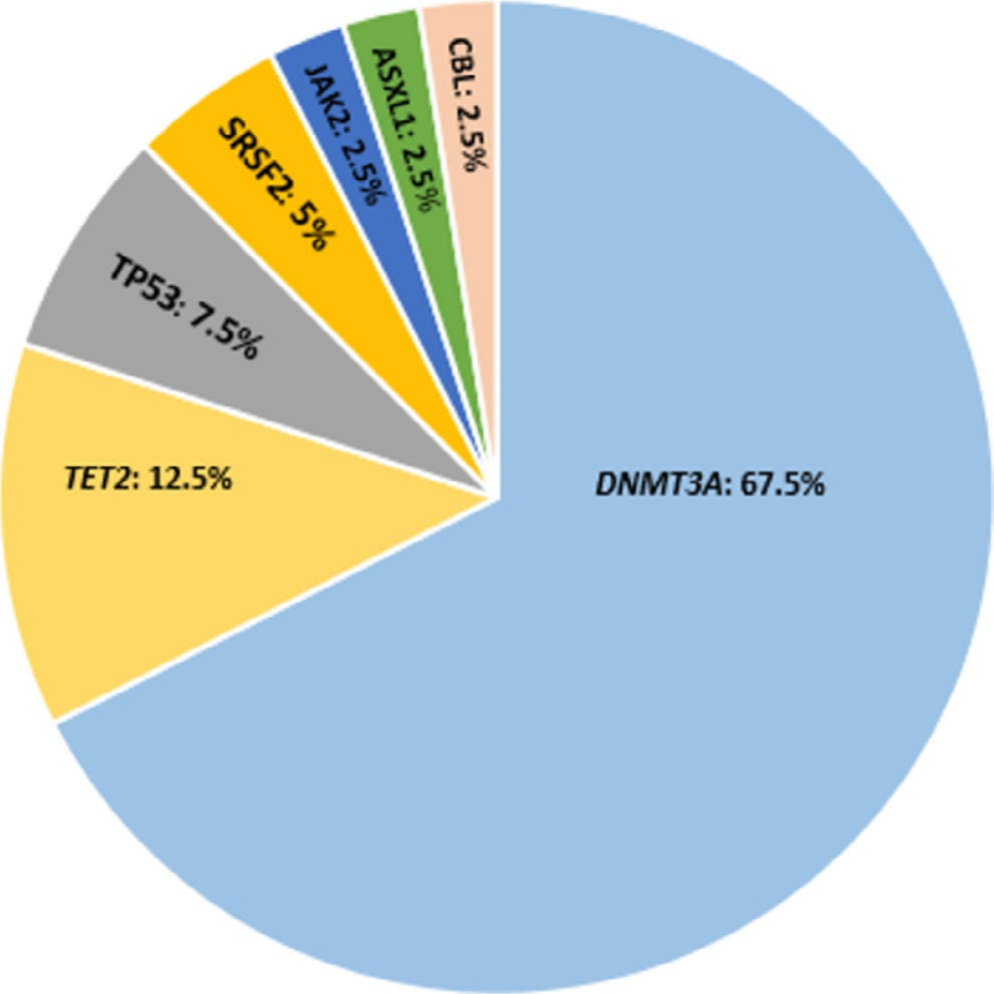
Outline of the somatic variants identified in the studied cohort. Distribution of pathogenic mutations with a VAF ≥ 0.5% in the overall cohort.

Molecular profiling with NGS analysis was performed in all the 39 patients. Selecting an initial, descriptive cut‐off of VAF at 0.5% for pathogenic/likely pathogenic variants, somatic mutations were detected in order of frequency in the following genes: *DNMT3A*, *TET2*, *TP53, JAK2 (non‐V617F), SRSF2*, *ASXL1*, *CBL* (Figure 2).

**FIGURE 3:**
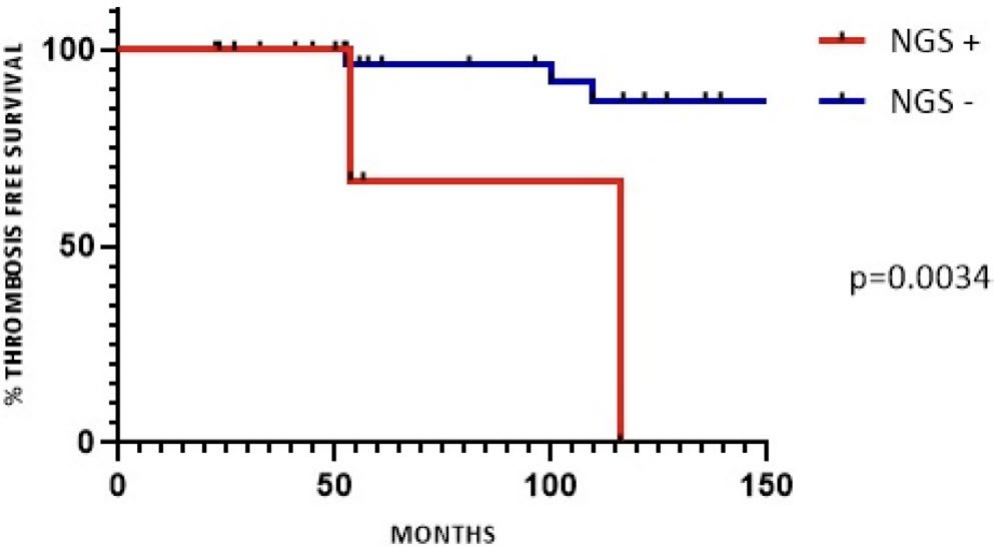
Thrombosis‐free survival in the studied cohort according to the molecular status. Thrombosis free survival was not reached in the NGS negative population (NGS‐) versus 116 months in the NGS positive (NGS+) population (*p*. 0.0034).

Filtering for pathogenic/likely pathogenic variants with a VAF of ≥ 5%, 6 patients (15.4%) resulted NGS‐positive, while 33 (84.6%) were NGS‐negative. In particular using this cut‐off, pathogenic mutations were detected in order of frequency in *DNMT3A* (4 patients, 7 variants), *TET2* (2 patients, 2 variants), *ASXL1* (2 patients, 2 variants) and *SRSF2* (1 patient, 1 variant). The two molecular groups showed significant differences in terms of mean age at diagnosis (65‐year‐old versus 43‐year‐old in the NGS positive vs. NGS negative patients, respectively; *p*. 0.01), IPSET‐score high (33.3% vs. 6.0% in the NGS positive vs. NGS negative patients, respectively; *p*. 0.04) and incidence of thrombotic events (TEs) (percentage of patients with ≥ 1 TE: 66.6% vs. 18.2% in the NGS positive versus NGS negative patients, respectively; *p*. 0.01). We observed a rate of 1.16 events per 100 patient‐years in the NGS‐negative cohort compared to 4 events per 100 patient‐years in the NGS‐positive population, during a median observation time of 54.4 and 145.2 months, respectively. The median thrombosis‐free survival (TFS) was not reached in the NGS negative group and was 116 months in the NGS positive group (Figure 3) (*p*. 0.0034). All the remaining clinical and laboratory features were similar in the two molecularly annotated cohorts. Likewise, the histopathological findings (i.e., consistent with ET versus not reaching the diagnostic criteria for ET) were similarly distributed within the two molecular groups. Table 1 summarizes the relevant clinical and laboratory data in the overall cohort and in NGS‐positive patients at VAF ≥ 5%.

To shed further insight into the thrombotic risk and associated risk factors, we compared the cohort of patients who experienced ≥ 1 TE (TE positive‐ TE^pos^) to the population of patients who never developed TE (TE negative‐ TE^neg^) (Table 2). A similar distribution of the main laboratory findings was also seen in the two cohorts, except for a higher occurrence of arterial hypertension (*p*. 0.03), dyslipidemia (*p*. 005) and IPSET‐high category (*p*. 0.0003) in TE^pos^ patients. Interestingly, the mean age at diagnosis was not different in the two groups. Instead, we confirm a significant association between NGS status and thrombotic risk: TE^pos^ patients were more likely to be NGS positive than NGS negative (40% vs. 6.9%, respectively; *p*. 0.01).

**TABLE 2:** Main clinical and laboratory findings in the investigated cohort, with stratification for history of thrombosis.

	TE^pos^: 10	TE^neg^: 29	*p*
Demographic parameter			
Male/Female, number of patients	3/7	20/9	ns
Age at diagnosis, mean (range)	50.4 (14–77)	49.3 (13–82)	ns
Laboratory values			
PLT count ×109/L, mean (range)	564 (493–767)	739 (481–1838)	ns
Hb (g/dL), mean (range)	13.4 (12.3–15.4)	13.8 (10.9–16.8)	ns
WBC count ×109/L, mean (range)	7.78 (4.75–10.04)	7.72 (5.2–11.00)	ns
LDH U/L, mean (range)	187 (145–228)	235 (145–313)	ns
EPO mU/mL, mean (range)	6.7 (7.2–9)	8.5 (2.3–25.8)	ns
Clinical findings			
Splenomegaly, number of patients	0 (0)	0 (0)	ns
IPSET‐low, number of patients (%)	2 (20)	23 (79.3)	0.0007
IPSET‐intermediate, number of patients (%)	4 (40)	6 (20.7)	ns
IPSET‐high, number of patients (%)	4 (40)	0 (0)	0.0003
Smoking, number of patients (%)	1 (10)	3 (10.3)	ns
Diabetes, number of patients (%)	0 (0)	0 (0)	ns
Hypertension, number of patients (%)	6 (60)	7 (24.1)	0.03
Dyslipidemia, number of patients (%)	5 (83.3)	7 (24.1)	0.005
Molecular findings			
NGS positive, number of patients (%)	4 (40)	2 (6.9)	0.01

*Note*: Data are split into two cohorts, according to history of thrombotic events: TEpos includes patients with reported TEs; TEneg includes patients with no history of TEs. A *p*. value ≤ 0.05 was considered significant.

Abbreviations: EPO, erythropoietin level; F, females; g/dL, grams per deciliter; Hb, hemoglobin; IPSET, International Prognostic Score of Thrombosis for Essential Thrombocythemia; LDH, lactate dehydrogenase; M, males; mU/mL, milliunits per milliliter; NGS‐neg, Next Generation sequencing negative patients; NGS‐pos, Next Generation sequencing positive patients; ns, not significant; PLT, platelet count; TEs, thrombotic events; U/L, unit per liter; yrs., year.

Variants with a VAF approaching 50% were detected in *JAK2, TET2*, and *SETBP1* (Table S2). From the clinical standpoint, none of these patients had a family history of thrombocytosis or of other hematological malignancies. However because the study was not designed to perform tumor‐normal paired testing, the presumed germline nature of these variants could not be assessed, and these were excluded from the final analysis.

Finally, a PLT count ≥ 900 × 10^9^/L was seen in 7 patients of the studied cohort. For all of them, NGS analysis did not detect any somatic mutations (using a VAF cut‐off of 5%). Interestingly, in spite of the severe thrombocytosis, none of these patients experienced TEs and/or hemorrhagic events at presentation or any time during follow‐up.

## 4 Discussion

In this real‐life, single‐center cohort study, we reviewed the clinical data and BMT biopsies of 39 consecutive TN‐ET and correlated them with the mutational profile at NGS.

We confirm that in the diagnostic workup of TN‐PT, NGS analysis should be performed in order to detect pathogenic non‐canonical myeloid mutations, perhaps with possible implications. In our cohort, using a cutoff of VAF of 5%, 15.4% of the cases resulted “clonal”.

Regarding the clinical behavior, we showed that TN‐PT is generally an indolent disease, with a virtually absent potential for evolution into secondary myelofibrosis and leukemic transformation. Nevertheless, the overall incidence of TEs in this condition was not negligible, as 25.6% of patients in our cohort experienced at least one thrombotic event. To investigate a possible role of NGS in identifying patients with different thrombotic risks, we generated two molecularly annotated cohorts, defined as “NGS positive” and “NGS negative” respectively. Compared to the NGS negative cohort, NGS positive patients were significantly more keen to develop TEs. On one side, we acknowledge that this result is partly influenced by an older age at diagnosis in the NGS positive population compared to the negative counterpart. Indeed, age is associated with an increased thrombotic risk and with the phenomenon of so‐called “clonal hematopoiesis” (CH). CH represents the accumulation over time of somatic mutations in hematopoietic stem cells of healthy individuals as a consequence of aging and has been associated with a pro‐inflammatory state linked to chronic conditions including cardiovascular diseases and thrombosis [20, 21]. CH is generally characterized by low burden variants, with higher burdens associated with increased probabilities to develop a clinically manifested disease [22]. Moreover, the most commonly mutated genes in CH are *DNMT3A, TET2, ASXL1* (so called “DAT” mutations) [20]. With the purpose of mitigating the effect of CH, we chose to set a VAF cut‐off at 5% for the selection of the variants to include in the analysis. Nevertheless, after filtering for this cut‐off, we identified pathogenic mutations almost exclusively on the DAT genes and, in patients developing TEs, only mutations on *DNMT3A* and *TET2*. This emphasizes how the clonal expansion of a previous smaller CH clone (i.e., with VAF < 5%) might contribute over time to produce an overt clinical pathologic phenotype.

To properly investigate risk factors associated with thrombosis in this cohort of TN‐PT, we compared the cohort of patients who experienced ≥ 1 TE (TE^pos^) to the population of patients who never developed TE (TE^neg^). Interestingly, age was not significantly different among the two groups, whilst NGS status itself was confirmed to represent a significant risk factor for thrombosis, as TE^pos^ patients were more likely to be NGS positive.

Because of the limited number of patients in our cohort, a multivariate analysis could not be performed. However, we can state that the identification of a clonal marker at NGS still represents an adjunctive risk factor for thrombosis, alongside other well‐established risk factors. Nevertheless, in our cohort of TN‐PT, TEs also occurred in patients who were NGS‐negative (6 patients of the overall cohort). In this subset, the mean age was 51 years (range 35–66 years), but the majority of these patients (83.4%) had an intermediate‐high IPSET score and were enriched for the presence of other common risk factors for vascular events. This underlines how the presence of a clonal marker represents only one of multiple predisposing factors for TEs, which act alongside the other well‐established prothrombotic triggers.

Regarding the histologic assessment, 60% of our cases displayed the characteristic histological features of ET. In terms of stromal alterations, a mild degree of reticulin fibrosis (“grade 1” as per WHO Classification grading system [6]) was seen in some cases, raising the issue of differential diagnosis with prefibrotic‐PMF (pre‐PMF); however, all the other histological features of pre‐PMF were not seen. Our study emphasizes the importance of performing a bone marrow biopsy in suspected PT, to investigate a possible MPN and to identify the specific MPN entity, especially in light of their highly diverse clinical behavior and prognosis [23]. We showed how, from the histological point of view, a subset (40% in our series) of patients with bona fide TN‐PT lack the classical microscopic features of ET or of other MPN. Of these, only two patients (14.3%) showed evidence of clonality at NGS analysis with pathogenic mutations affecting *DNMT3A* and *TET2*. We did not find non‐canonical driver mutations previously associated with MPN diseases in these patients. In similar cases, lacking the histopathologic findings of ET (which is one of the requested findigs for this diagnosis), the presence of a clonal marker can at least support a primary rather than reactive nature of thrombocytosis.

Other studies have addressed the topic of TN‐ET and PT. In a cohort of 40 patients with TN‐ET, using a VAF cut‐off of ≥ 3%, Cattaneo et al. have detected variants in 87.5% of the cases. The majority of patients showed histologic findings in keeping with ET. The overall incidence of TEs was 5%, and no leukemic evolutions were reported [24]. In a cohort of 266 consecutive cases of ET, Santoro M. et al. identified 16.9% of patients with a TN molecular phenotype. Comparing the clinical outcome of the mutated cohort to that of the TN one, no significant differences were noted in risk of TEs, incidence of progression to secondary myelofibrosis or BP, and overall survival; TN patients were instead significantly younger at the time of first diagnosis, as seen in our cohort, and presented with a lower symptom burden [5]. In a study by Michail et al. [25], setting a cut‐off at 5% for VAF, 11.1% of the patients showed at least one mutation. TEs prior to and following the diagnosis of TN‐PT were noted in 20% and 17% of patients, respectively. No patients progressed to secondary myelofibrosis or BP 25. Lastly, Lemoine et al. analyzed a cohort of 130 patients with TN thrombocytosis. Around 60% of patients met the criteria for a MPN/ET. Using a VAF cut‐off at 2%, at least one pathogenic somatic mutation was detected in 29.2% of cases. The presence of pathogenic variants was associated with an increased tendency of thrombotic events [4].

Our results present some limitations deriving from a numerically limited and monocentric population and would need to be validated in larger, possible prospective cohorts. We believe that this limitation can hardly be avoided when dealing with rare conditions, such as TN‐PT. Moreover, we have applied a targeted NGS gene panel which, albeit designed to test commonly mutated genes in myeloid malignancies, provides information only on a limited amount of genes. In this context, and especially in patients with no evidence of clonality using the commercially available targeted panels, more extensive testing techniques, as whole exome sequencing, would be more informative. Lastly, we could not prove the possible germline origin in variants with VAF approaching 50%, which could represent a limit of our study.

In spite of such limitations, our data highlight that TN‐PT comprises a heterogeneous group of patients with different molecular backgrounds, thrombotic risk, and an overall indolent behavior in terms of fibrotic progression and leukemic evolution. The use of targeted NGS analyses can show evidence of clonality and helps stratify patients with different thrombotic risks, thus providing useful clinical information.

## Author Contributions

Valentina Sangiorgio and Elena Maria Elli have designed the study and produced the main manuscript. Valentina Sangiorgio and Fabio Pagni have blindly reviewed the bone marrow trephine biopsies. Federica Mottadelli, Martina Venegoni and Giovanni Cazzaniga have performed the molecular studies. Fabrizio Cavalca has performed the statistical analyses and collected the clinical data. Rocco Piazza and Carlo Gambacorti‐Passerini have overviewed the study.

## Ethics Statement

The authors have nothing to report.

## Consent

An Informed consent specific for the current study was obtained for all the enrolled patients. This included the possibility to collect clinical information and molecular (i.e., somatic only) data from blood or bone marrow samples.

## Conflicts of Interest

The authors declare no conflicts of interest.

## Supporting information


**Data S1.** Supporting Information.

## Data Availability

The data that support the findings of this study are available on request from the corresponding author. The data are not publicly available due to privacy or ethical restrictions.
