# Supplementary material for: Next Generation Sequencing Identifies Subgroups of Patients With Triple Negative Primary Thrombocytosis With Different Clinical Thrombotic Outcomes
Source: Int J Lab Hematol. 2025 Apr 7;47(5):869–76. doi: 10.1111/ijlh.14476 (PMC12426806; doi:10.1111/ijlh.14476)
Supplement: Supplementary file 1 — Data S1. Supporting Information. [file IJLH-47-869-s001.docx]

**Supplementary material.**

**Supplementary table 1: List of genes analyzed within the SOPHiA GENETICS™ Myeloid Solution.**

*ABL1* (4-9), *ASXL*1 (10,12,13), *BRAF* (15), *CALR* (9), *CBL* (8,9), *CEBPA* (all), *CSF3R* (all), *DNMT3A* (all), *ETV6* (all), *EZH2* (all), *FLT3* (13-15,20), *HRAS* (2,3), *IDH1* (4), *IDH2* (4), *JAK2* (all), *KIT* (2,8-11,13,17,18), *KRAS* (2,3), *MPL* (10), *NPM*1 (10,11), *NRAS* (2,3), *PTPN11* (3,7-13), *RUNX1* (all), *SETBP1* (4), *SF3B1* (10-16), *SRSF2* (1), *TET2* (all), *TP53* (all), *U2AF1* (2,6), *WT1* (6-10), *ZRSR2* (all).

Reference: <https://www.sophiagenetics.com/clinical/oncology/blood-cancers/sophia-ddm-dx-myeloid-solution>.

**Supplementary table 2: Description of identified variants.**

Legend: AA, amino acid; B, benign; C, controversial; CDS, coding DNA sequence; LB, likely benign; LP, likely pathogenic; na, not available; P, pathogenic; VUS, variant of unknown significance.

*Variant classification according to the following clinical databases: 1) Varsome (https://varsome.com); 2) Franklin by Genoox (https://franklin.genoox.com/clinical-db/home); 3) Clinvar (https://www.ncbi.nlm.nih.gov/clinvar). In silico predictions have been reported querying the following predictors: FATHMM-MKL (https://fathmm.biocompute.org.uk/fathmmMKL.htm); DANN (Quang D, Chen Y, Xie X. DANN: a deep learning approach for annotating the pathogenicity of genetic variants. Bioinformatics. 2015;31(5):761-763); SIFT (https://provean.jcvi.org/index.php). In italics are variants with a VAF approaching 50%, the possible germline origin of which could not be tested.
